# Incidence, clinical characteristics, and prognostic nomograms for patients with myeloid sarcoma: A SEER-based study

**DOI:** 10.3389/fonc.2022.989366

**Published:** 2022-08-18

**Authors:** Ziping Xing, Xiaohua Zhu, Zifeng Li, Hongsheng Wang, Maoxiang Qian, Xiaowen Zhai

**Affiliations:** ^1^ Department of Hematology and Oncology, National Children’s Medical Center, Children’s Hospital of Fudan University, Shanghai, China; ^2^ Institute of Pediatrics, National Children’s Medical Center, Children’s Hospital of Fudan University, Shanghai, China

**Keywords:** SEER, myeloid sarcoma, nomogram, prognosis, hematopoietic site

## Abstract

**Background:**

Myeloid sarcoma (MS) is a rare hematological tumor that presents with extramedullary tumor masses comprising myeloid blasts. A controversial issue is whether MS involving normal hematopoietic sites (liver, spleen, and lymph nodes) should be excluded in future studies. We aimed to compare MS characteristics and outcomes involving hematopoietic and non-hematopoietic sites and construct a prognostic nomogram exclusively for the latter.

**Methods:**

Data from patients diagnosed with MS between 2000 and 2018 were collected from the Surveillance, Epidemiology, and End Results (SEER) database. According to the primary site, patients were classified as having MS involving hematopoietic sites (hMS) or non-hematopoietic sites (eMS). Clinical characteristics and survival outcomes were compared between the two groups using Wilcoxon, chi-square, and log-rank tests. Cox regression analysis was used to identify eMS prognostic factors to establish prognostic nomograms. The models’ efficiency and value were assessed using receiver operating characteristic (ROC) curves, calibration curves, and decision curve analysis (DCA).

**Results:**

In total, 694 patients were enrolled, including 86 with hMS and 608 with eMS. There were no sex, race or marital status distribution differences between the two groups. Patients with eMS had better overall and cancer-specific survival rates than those with hMS. Additionally, prognostic factor effects differed between the two groups. Patients with eMS were randomly divided into the training (number of patiens, n=425) and validation cohorts (n=183). Age, first primary tumor, primary site, and chemotherapy were used to establish nomograms. The C-index values of overall survival (OS) and cancer-specific survival (CSS) nomograms were 0.733 (validation: 0.728) and 0.722 (validation: 0.717), respectively. Moreover, ROC, calibration curves, and DCA confirmed our models’ good discrimination and calibration ability and potential clinical utility value.

**Conclusion:**

Our study described the differences between patients with eMS and those with hMS. Moreover, we developed novel nomograms based on clinical and therapeutic factors to predict patients with eMS’ 1-, 3- and 5-year survival rates.

## Introduction

Myeloid sarcoma (MS), a term that accurately summarizes the two features of this disease, is a rare hematologic tumor composed of myeloid cells in bone, soft tissues and other anatomical sites (1). Due to this entity’s rarity, much of our current limited MS clinical and prognostic characteristics understanding is derived from case reports or single-center studies. In addition, several terms are used in clinical diagnoses and academic reports to describe MS, including chloroma, granulocytic sarcoma, and extramedullary acute myeloid leukemia (eAML) (1–3). The confusion over MS terminologies has further impeded this disease’s comprehensive study, especially its epidemiological features.

Other important MS features are that it can occur at any site of the body, except the bone marrow, and present synchronously or subsequently with various myeloid malignancies, including acute myeloid leukemia (AML), myelodysplastic syndrome (MDS), myeloproliferative neoplasm (MPN), or chronic myelogenous leukemia (CML) (4–6). Because infiltration of leukemic cells in the liver, spleen or lymph nodes is generally considered to be an indication of the natural spread of tumor cells from the bone marrow, several scholars have argued that myeloid neoplasms originating from normal hematopoietic sites should not be included in MS (5, 7). The term “extramedullary acute myeloid leukemia (eAML)” was proposed to describe MS involving non-hematopoietic sites in their studies (8). However, excluding myeloid masses involving normal hematopoietic sites from MS is based only on theoretical derivation. The differences between patients with MS involving normal hematopoietic and non-hematopoietic sites have yet to be illustrated in the literature.

The Surveillance, Epidemiology, and End Results (SEER) database, a population-based oncology clinical database in the United States, provides a wealth of information for research on rare tumors. However, the latest published literature on MS, based on the SEER database, only included patients (≥15 years old) from 1973 to 2010 (9). We enrolled patients diagnosed with MS between 2000 and 2018 from the SEER database and divided them into two categories: MS involving hematopoietic sites (hMS) and those involving non-hematopoietic sites (eMS). We aimed to update our understanding of MS regarding its epidemiological, clinical, and prognostic characteristics, describe the differences between patients with eMS and hMS, and further develop MS prognostic nomograms.

## Materials and methods

### Patient selection and data collection

All data involved in this study were obtained from the SEER database software (SEER*Stat version 8.4.0). Age-adjusted rates and trends in rates of MS from 2000 to 2018 were calculated in the rate session. MS patient selection and clinical data collection were carried out in the case listing session based on the SEER reseach dataset (18 registries, [2000-2018]). As shown in the flow chart **(** **Figure S1** **)**, the inclusion criteria of MS patients were as follows: (1) the International Classification of Disease for Oncology, Third Edition (ICD-O-3) histology code 9930/3; (2) positive exfoliative cytology or positive histology diagnosis. The exculsion criteria were as follows: (1) clinical diagnosis, image diagnosis, or unknown diagnosis; (2) primary site labels C42.0 Blood, C42.1 Bone marrow, or unknown site. To describe the clinical characteristics of MS patient, the following clinical information was extracted: age and marital status at diagnosis, sex, race, year of diagnosis, total number of tumors per patient, first malignant primary indicator, primary site, treatment (surgery, radiotherapy, and chemotherapy), survival time, survival status, and cause of death. SEER is a free and publicly available database and has anonymized the patient’s identifying information. Therefore, there are no ethical issues, and approval from the ethics committee was not required.

According to anatomic sites, patients were classified into two groups: MS involving hematopoietic sites (hMS) and those involving non-hematopoietic sites (eMS). Hematopoietic sites include the spleen, liver and lymph nodes. According to the classification principles proposed by Goyal et al., non-hematopoietic sites can be further divided into 9 major categories as follows: soft tissue(st), skin/breast (s/b), bone (b), nervous system (ns), head/neck (h/n), digestive system(ds), cardiopulmonary/mediastinum (c/m), reproductive system (rs), and kidney/bladder/retroperitoneum (k/b/r) **(** **Table S1** **)** (10). Furthermore, patients with eMS were randomly divided into a training and a validation cohort by a ratio of 7:3 to develop prognostic prediction models. This study’s two endpoints, overall survival (OS) and cancer-specific survival (CSS), were defined as the time from the initial diagnosis to death related to any cause and MS, respectively.

### Construction, validation and evaluation of nomograms

Univariate and multivariate COX regression analyses were performed to identify the independent risk factors for OS and CSS of patients with eMS in the training cohort. Moreover, the C-index and Akaike information criterion (AIC) were calculated to determine the final independent prognostic factors for inclusion in the prognostic nomograms. Regarding the performance of the prognostic nomograms, the area under the receiver operating curve (AUC) was calculated to examine the discrimination power; calibration curves were plotted to test the predictive accuracy; and decision curve analysis (DCA) was used to evaluate the clinical utility. Based on the nomograms, each patient was assigned a total point to predict survival rates. As the scores rise, the survival rates fall. Then, the best cutoff values for the total points were generated using X-tile software and used for risk stratification (low and high). To evaluate the significance of the OS and CSS differences between the low- and high-risk groups, we also conducted Kaplan-Meier survival analysis and log-rank tests.

### Statistical analysis

Continuous variables were compared between groups using the Mann-Whitney U-test, whereas categorical variables were compared using the chi-squared test. Survival outcomes between the groups were visualized using Kaplan–Meier curves and compared using a log-rank test. Overall, statistical analyses in this study were conducted using R software (version 4.0.5) with the “survival,” “survminer,” “rms,” and “ggDCA” packages. P-value <0.05 was considered statistically significant.

## Results

### Incidence trends of MS

**Figure 1:**
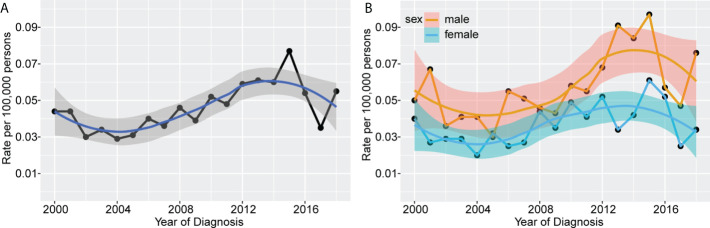
Incidence rates of MS according to year of diagnosis. The waveringly increasing trends were observed in incidence rates of all patients with MS from 2000 to 2018 **(A)**, and patients grouped by sex **(B)**.

We used the SEER database to calculate age-adjusted MS incidence rates by year of diagnosis and gender. A rising pattern in MS incidence from 2000 to 2018 was observed, with an annual percentage change (APC) of 3.20% (95% confidence interval [CI]: 1.21–5.23%, P=0.003) **(** **Figure 1A** **).** After peaking at 0.077 per 100,000 persons in 2015, the age-adjusted MS incidence plummeted to 0.035 per 100,000 persons in the following two years. Grouped by sex, we found the waveringly increasing trend was more noticeable in male patients, with an APC of 3.44% (95%CI 0.99-5.96%, P=0.008) and 2.65% (95%CI 0.14-5.23%, P=0.039) in male and female patients, respectively **(** **Figure 1B** **)**. In general, male patients had a substantially higher incidence than females.

### Clinical characteristics of patients

We identified 694 patients with MS from the SEER database between 2000 and 2018 and summarized their demographic and clinical characteristics in **Table 1**. The median age of patients with MS in this study was 62 years, ranging from 0 to 96 years. Most patients were: aged above 60 years (53.2%), male (57.5%), white (80.1%), married (49.7%), and diagnosed between 2010 and 2018 (63.7%). In addition, most had MS as the first primary tumor (56.8%). Furthermore, half of the patients (49%) underwent chemotherapy, whereas only a small proportion underwent radiotherapy (24.9%) or surgery (13.1%).

**Table 1:** Baseline characteristics of patients diagnosed with MS from the SEER database.

Characteristic	eMS	hMS	Total	P.Value
		N(%)	N(%)		N(%)	
Sum	608(87.6)	86(12.4)	694(100)	
Age at diagnosis (years)				0.011
Mean (SD)	55.5(22.5)	61.7 (19.7)	56.2(22.3)	
Median [Min, Max]	61.0 [0, 96.0]	67.5 [3,91.0]	62.0 [0, 96.0]	
Age group (years)				0.149
<40	140 (23.0)	14 (16.3)	154 (22.2)	
40-59	153 (25.2)	18 (20.9)	171 (24.6)	
≥60	315 (51.8)	54 (62.8)	369 (53.2)	
Year of diagnosis				0.513
2000-2009	224 (36.8)	28 (32.6)	252 (36.3)	
2010-2018	384 (63.2)	58 (67.4)	442 (63.7)	
Sex				0.826
Female	257 (42.3)	38 (44.2)	295 (42.5)	
Male	351 (57.7)	48 (55.8)	399 (57.5)	
Race				0.282
Asian	47 (7.7)	5 (5.8)	52 (7.5)	
White	490 (80.6)	66 (76.7)	556 (80.1)	
Others	71 (11.7)	15 (17.4)	86 (12.4)	
Marital.status				0.096
Single	151 (24.8)	18 (20.9)	169 (24.4)	
Married	304 (50.0)	41 (47.7)	345 (49.7)	
Widowed	49 (8.1)	14 (16.3)	63 (9.1)	
Others	104 (17.1)	13 (15.1)	117 (16.9)	
Number				0.074
≥3	94 (15.5)	19 (22.1)	113 (16.3)	
1	265 (43.6)	42 (48.8)	307 (44.2)	
2	249 (41.0)	25 (29.1)	274 (39.5)	
1^st^ primary tumor				0.875
No	264 (43.4)	36 (41.9)	300 (43.2)	
Yes	344 (56.6)	50 (58.1)	394 (56.8)	
Surgery				0.103
No/Unknown	523 (86.0)	80 (93.0)	603 (86.9)	
Yes	85 (14.0)	6 (7.0)	91 (13.1)	
Radiation				0.008
No/Unknown	446 (73.4)	75 (87.2)	521 (75.1)	
Yes	162 (26.6)	11 (12.8)	173 (24.9)	
Chemotherapy				0.205
No/Unknown	310 (51.0)	37 (43.0)	347 (50.0)	
Yes	298 (49.0)	49 (57.0)	347 (50.0)	

As previously stated, patients were divided into two groups depending on the site involved: hMS (n=86) and eMS (n=608). A comparison of the two groups’ demographic and clinical features is shown in **Table 1**. Except for age and radiotherapy proportion received, there were no statistical differences in the other variables between the eMS and hMS groups. Specifically, the hMS group had a higher patient proportion aged >60 years, with a median age of 67.5 years. Regarding treatment, patients with hMS were more likely to undergo chemotherapy (57% vs. 49%, P=0.205) and were less likely to undergo surgery (7% vs.14%, P=0.103) and radiotherapy (12.8%vs. 26.6%, P=0.008) than patients with eMS. In addition, the three most common involvement sites were soft tissues (35.6%), skin/breast (13.0%), and the digestive system (9.4%) (**Table S1**). The mean age of patients with eMS involving the digestive system, reproductive system, and head/neck was lower (<50 years) than that of the patients with eMS involving other sites (>50 years).

In consideration that pediatric patients were not included in the previous SEER-based study, we analyzed MS clinical characteristics in pediatric patients aged <15 years old (9). Among the 694 patients with MS, only 47 pediatric patients were identified. As shown in **Table S2**, pediatric patients’ mean age was 5 years. Furthermore, 87.2% of children developed MS as the first primary tumor, which is far more common than in adults. In addition, the three most common sites in pediatric patients differed slightly from those in adults, with the head/neck rather than the digestive system being one of our study’s three most common sites. Nearly three-quarters of pediatric patients receive chemotherapy, higher than the 50% in adults. Overall, pediatric patients’ prognosis was also good, with a 3-year OS rate of 67.4%.

### Survival analysis

**Figure 2:**
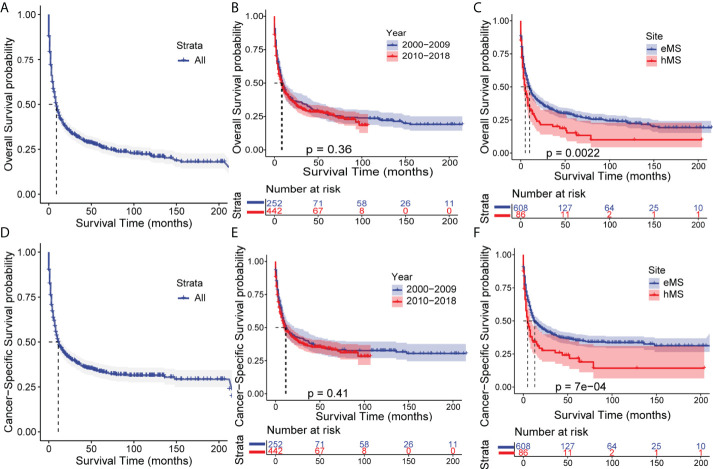
Kaplan–Meier analysis of overall survival (OS) and cancer-specific survival (CSS) in MS. Kaplan–Meier survival curves of OS for all patients **(A)**, patients stratified by year of diagnosis **(B)** and primary sites **(C)**. Kaplan–Meier survival curves of CSS for all patients **(D)**, patients stratified by year of diagnosis **(E)** and primary sites **(F)**. eMS, MS excluding those involving hematopoetic sites; hMS, MS involving hematopoetic sites.

Of the 694 patients with MS in this study, 498 died (71.76%); 410 died of MS (59.08%). We performed a Kaplan-Meier survival analysis to quantify and visualize OS and CSS in patients and used the log-rank test to compare survival outcomes between patients grouped by year of diagnosis and primary sites. As shown in **Figure 2**, the median OS time for patients with MS was 9 months, with a 31.3% 3-year OS rate. The median CSS time was 11 months, with a 37.7% 3-year CSS rate. Despite the growing number of patients, the OS and CSS of patients diagnosed in the last decade (2010-2018) did not differ from those in the previous decade (2000-2009), showing no significant improvement in survival outcomes over the last two decades. Additionally, patients with hMS had significantly lower OS and CSS rates than those with eMS, with a median OS of 5 and 10 months and median CSS of 5 and 13 months, respectively (**Table S1**).

**Figure 3:**
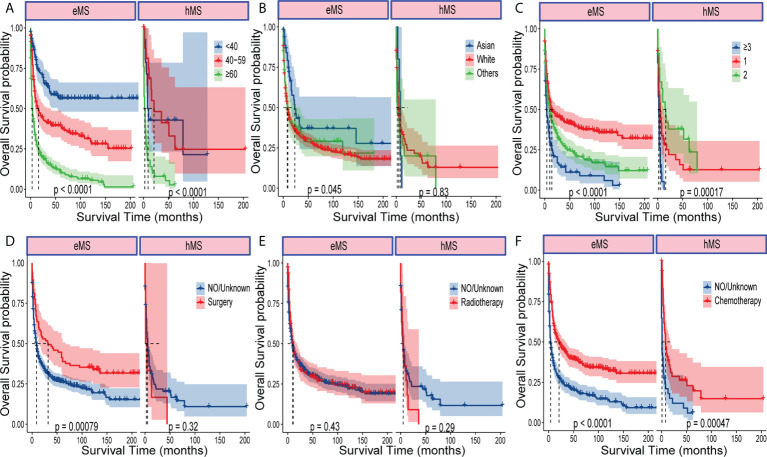
Kaplan–Meier analysis of OS in eMS and hMS, respectively. Kaplan–Meier survival curves of OS for patients with eMS or hMS stratified by age **(A)**, race **(B)**, number of tumors **(C)**, surgery **(D)**, radiotherapy **(E)** and chemotherapy **(F)**.

We performed a subgroup survival analysis to determine whether different variable prognostic effects were consistent between hMS and eMS. As shown in **Figure 3** and **Figure S2**, age, race, and tumor number had different effects on eMS and hMS prognosis. Given that the number of patients undergoing surgery and radiotherapy in the hMS group was too small, we should be cautious when concluding how treatment affects prognosis. The fact that the variables had different prognostic effects between the two groups suggested that hMS should be distinguished from eMS in future studies.

According to the 3-year OS rate shown in **Table S1**, the nine primary site categories in patients with eMS were further divided into four sets for the prognosis analysis as follows: setA (3-year OS <25%; nervous system and bone), setB (3-year OS: 25%–35%; cardiopulmonary/mediastinum, kidney/bladder/retroperitoneum, and soft tissue), setC (3-year OS: 35%–50%; skin/breast, head/neck, digestive system), and setD (3-year OS >50%; reproductive system). Patients with setA had the poorest prognosis among the four sets, and those with setD had the best prognosis.

### Prognostic factors selection and nomograms construction

**Figure 4:**
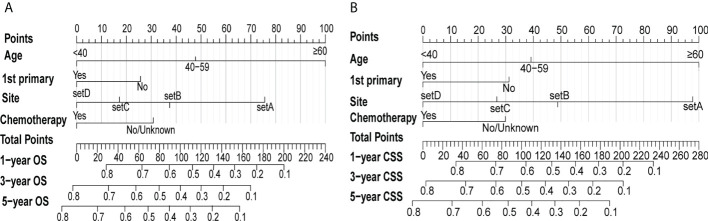
Nomograms of predicting the 1-, 3- and 5-year OS rates **(A)** and CSS rates **(B)** for eMS patients. The first line called “Points” is the score reference of the 4 variables below. For any given patient, the score of each variable can be obtained by drawing a vertical line from the corresponding scale axis to the first line “Points”. Then, sum up the 4 scores to obtain the total points, which can be mapped to predict 1-, 3-, and 5-year survival rates by drawing a line descending from the axis labeled “Total points” to the 3 survival axes.

Patients with eMS were randomly divided into the training (n=425) and validation cohorts (n=183). Patients in the training cohort were screened for independent prognostic markers. None of the eleven variables included in the univariate COX analysis was statistically different between the training and validation cohorts (**Table S3**). The univariate Cox analysis showed that the year of diagnosis, sex, race, and radiotherapy were not significantly associated with the OS and CSS of patients with eMS (**Table S4**). As shown in **Table 2**, we included the remaining seven variables in the multivariate Cox analysis and demonstrated that marital status, tumor number, and surgery were not significant risk factors for OS and CSS (P >0.05). Patients aged >40 years, diagnosed with MS involving the nervous system or bone and not as 1^st^ primary tumor, and who did not undergo chemotherapy had worse survival outcomes (HR>1, P <0.05). Combining the multivariate Cox regression analysis results and AIC, we eventually identified age (<40, 40-59, ≥60), first primary tumor (yes, no), site (setA, B, C, D), and chemotherapy (no/unknown, yes) as significant prognostic factors to construct OS and CSS nomograms (**Figure 4**). The OS and CSS nomograms’ C-index values were 0.733 (95%CI: 0.703-0.762) and 0.722 (95%CI: 0.698-0.755) in the training cohort, 0.728 (95%CI: 0.679-0.757) and 0.717 (95%CI: 0.664-0.770) in the validation cohort, respectively.

**Table 2:** Multivariate COX regression analyses of OS and CSS in the training cohort.

Characteristics	OS				CSS	
		HR	95%CI	P.Value	HR	95%CI	P.Value
Age*						
<40	Ref			Ref		
40-59	2.17	1.39-3.40	<0.001	1.82	1.12-2.96	0.015
≥60	4.21	2.69-6.58	<0.001	3.57	2.21-5.76	<0.001
Marital.status						
Single	Ref			Ref		
Married	0.80	0.55-1.17	0.251	0.76	0.50-2.25	0.199
Widowed	1.34	0.80-2.24	0.271	1.20	0.68-2.13	0.529
Others	0.91	0.59-1.39	0.654	0.84	0.53-1.34	0.461
Number						
≥3	Ref			Ref		
1	0.98	0.60-1.58	0.929	0.86	0.51-1.47	0.589
2	0.84	0.61-1.17	0.311	0.74	0.52-2.06	0.099
1^st^ Primary Tumor*						
No	Ref			Ref		
Yes	0.67	0.46-0.98	0.038	0.69	0.45-1.05	0.086
Site*						
SetA	Ref			Ref		
SetB	0.62	0.42-0.90	0.013	0.58	0.38-0.87	0.009
SetC	0.48	0.32-0.72	<0.001	0.45	0.29-0.71	<0.001
SetD	0.43	0.23-0.80	0.008	0.38	0.18-0.77	0.007
Surgery						
No/Unknown	Ref			Ref		
Yes	0.73	0.51-1.05	0.091	0.75	0.49-1.12	0.161
Chemotherapy*						
No/Unknown	Ref			Ref		
Yes	0.65	0.51-0.84	<0.001	0.70	0.53-0.93	0.012

OS, overall survival; CSS, cancer-specific survival; HR, hazard ratio; CI, confidence interval; setA, nervous system and bone; setB, cardiopulmonary/mediastinum, kidney/bladder/retroperitoneum and soft tissue; setC, skin/breast, head/neck, and digestive system; setD, reproductive system. The symbol *indicates that the variable is statistically significantly in the COX regression analysis with P. value < 0.05.

### Nomogram validation and evaluation

**Figure 5:**
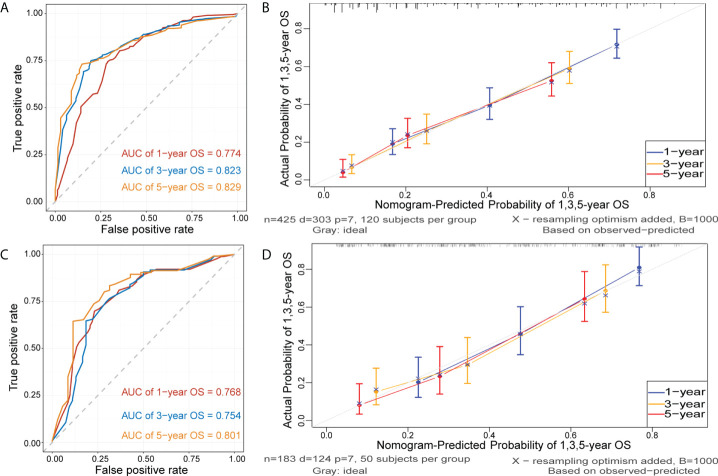
Receiver operating characteristic (ROC) and calibration curves of the nomogram for OS. ROC curves were plotted to evaluate the performance of the model to discriminate between patients with different survival outcomes (alive or dead), quantified by calculating the area under the ROC curve (AUC). The AUC of nomogram for predicting 1-, 3-, and 5-year OS were 0.774, 0,823 and 0.829 in the training cohort **(A)** and 0.768, 0.754, and 0.801 in the validation cohort **(C)**. Calibration curves were plotted to evaluate the accuracy of the nomogram model. The horizontal axis represents the survival rate predicted by the model, and the vertical axis represents the actual survival rate. The diagonal line represents the ideal situation where the predicted and actual survival rates consist, and the blue, orange, and red lines represent the model’s predicted and actual survival rates for 1-year, 3-year, and 5-year OS, respectively. Calibration curves of the nomogram for predicting 1-, 3-, and 5-year OS in the training cohort **(B)** and in the validation cohort **(D)**.

**Figure 6:**
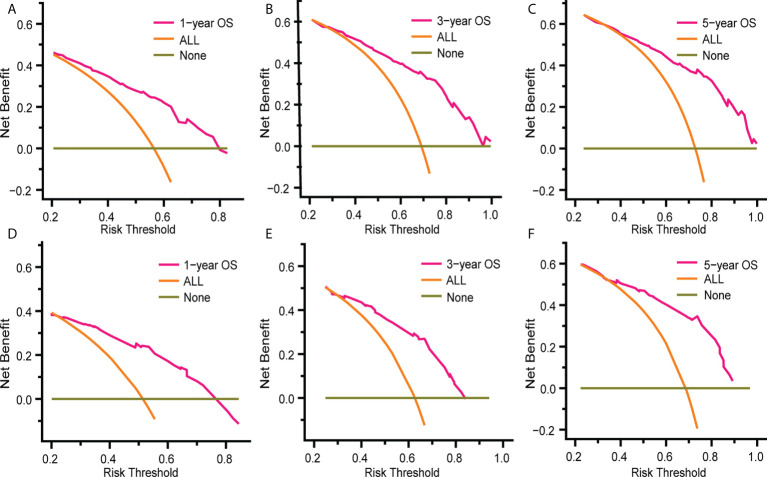
Decision curve analysis (DCA) of the nomogram for predicting 1-, 3- and 5- year OS rates in patients with eMS in the training cohort **(A–C)** and the validation cohort **(D–F)**. DCA was used to evaluate the clinical utility of the nomogram by calculating the net benefits of the model under different thresholds. The x-axis represents threshold probability, and the y-axis represents net benefit. The horizontal dark green line represents no deaths occurring, and the orange line represents all patients died. The pink line represents our nomogram model and when it is maintained above the dark green and orange line mentioned above, the net benefit value of the model is positive, which implies that our model has good clinical utility.

**Figure 5** and **Figure S3** show that time-dependent receiver operating characteristic (ROC) and calibration curves were drawn to evaluate OS and CSS nomograms’ discrimination and calibration ability, respectively. The AUC was calculated to assess the performance. The OS and CSS nomograms presented an AUC value of 0.774-0.823-0.829 and 0.758-0.812-0.822 for the training cohort’s 1-, 3-, and 5-year survival rates, and 0.768-0.754-0.801 and 0.755-0.756-0.811 for the validation cohort’s 1-, 3-, and 5-year survival rates. In addition, the calibration curves revealed outstanding consistency between actual survival rates and nomogram-predicted survival rates at 1, 3, and 5 years in both the training and validation cohorts. DCA was used to assess the nomograms’ clinical utility. **Figure 6** and **Figure S4** showed that the OS and CSS nomograms had a major positive net benefit, indicating good clinical utility and favorable efficiency in predicting 1-, 3-, and 5-year survival rates.

**Figure 7:**
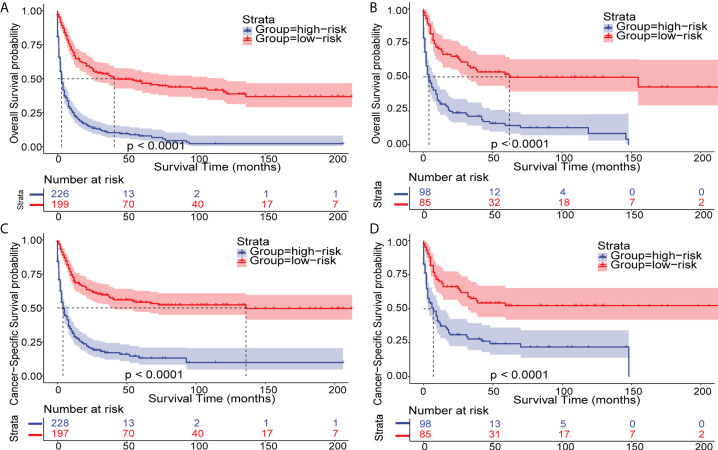
Kaplan–Meier analysis of OS and CSS in patients with eMS. Kaplan–Meier survival curves of OS and CSS in eMS patients stratified by risk levels in the training cohort **(A, C)** and validation cohort **(B, D)**.

To extend the nomograms’ clinical application, we stratified patients into two groups based on their nomogram points: high-risk with higher points and low-risk with lower points. The best cutoff values for OS and CSS nomogram points were 139.3 and 130.7, respectively (**Figure S5**). In general, Kaplan-Meier survival analysis revealed that patients with eMS could be classified into low-risk patients with a better prognosis and high-risk patients with a worse prognosis (P<0.0001, **Figure 7**).

## Discussion

Given MS’s rarity and terminology confusion, most of its knowledge is based on a case series of single-center studies, wherein the retrospective analysis of a limited number of patients may lead to conflicting findings (7, 11). Although a previous MS study using the SEER database was published, it only covered adult patients aged >15 years from 1973 to 2010 (9). MS’s epidemiological, clinical, and prognostic characteristics are poorly understood. Our study updates MS understanding based on the SEER database by including patients from 2000 to 2018 and provides MS epidemiological features and prognostic nomograms.

As previously stated, MS was listed as an AML subtype by the World Health Organization (WHO) and considered as a specific presentation of many other myeloid neoplasms, including MDS, MPN, and CML (1, 12, 13). Recently, two cases were reported in which MS and acute lymphoblastic leukemia occurred simultaneously (14, 15). MS’s complex association with other hematologic neoplasms has resulted in a lack of separate epidemiologic data. Previous studies have only focused on the patient proportion who presented with MS in AML and showed that the MS occurrence rate in AML was 2-9% in adults and 6.8-23.3% in children (2, 9, 10, 16, 17). Our study is the first to report the MS age-adjusted incidence among the US population from 2000 to 2018. We showed a fluctuating increasing trend in the incidence rate and observed a peak in incidence in 2015, one year before the WHO re-adopted the term MS (4). Consistent with the higher male patient proportion, their incidence rate was notably higher than that of female patients. However, since MS is easily ignored or misdiagnosed in clinical practice, with up to 50% of cases being misdiagnosed as lymphoma or Ewing sarcoma, the actual prevalence may be underestimated (18, 19).

In this study, we comprehensively analyzed MS’s demographic and clinical characteristics. The patients were predominantly older Caucasian men, consistent with previous studies based on the SEER and NCDB databases (9, 10). Our study analyzed tumor numbers in patients with MS and whether MS was the first primary tumor. As reported in previous studies, nearly 80-90% of newly diagnosed patients with MS may have an AML or other hematological neoplasm history, either at diagnosis or later in the disease course (7, 20). However, patients with MS as the initial and only tumor, also known as isolated MS, accounted for nearly half of the patients in our study, suggesting that isolated MS was more prevalent than MS combined with other hematologic malignancies. Understanding of MS in association with other hematologic cancers is improving. However, there is still more to learn about isolated MS (21).

MS can occur at any extramedullary anatomical site; however, the primary site classification principles have not been standardized. The orbit and gonads, considered privileged sanctuary sites with a better prognosis, were highlighted as separate categories in previous SEER-based studies (9). The primary site classification in the NCDB-based study, which integrates anatomical locations, organ systems, and prognosis, was adopted in this study (10). As previously described, we grouped the lymph nodes, spleen, and liver as normal hematopoietic sites, whereas the remaining non-hematopoietic sites were divided into nine categories and then grouped into four sets based on the 3-year OS. We found that the most common sites were soft tissue, skin/breast, and digestive system, consistent with NCDB- and SEER-based study results. In addition, numerous studies have identified the primary site as an independent prognostic factor (7, 22). In our study, patients with MS involving the head/neck, reproductive system, or digestive system had a better prognosis. However, the better prognosis could be partly due to the younger age of patients with MS involving these sites.

According to Shallis et al., several scholars recommend distinguishing patients with hMS from those with eMS, stating that myeloid blasts involving normal hematopoietic sites should be diagnosed as extramedullary leukemia infiltrates, rather than MS (8, 22, 23). However, apart from the distinct onset sites, the differences between these two concerning critical elements, including clinical and molecular characteristics, have not been published in the literature. In this study, we demonstrated demographic and clinical characteristic differences between patients with hMS and those with eMS. On average, patients with hMS were older than those with eMS, with a higher proportion of patients receiving local therapy. We also observed that the effects of age, race, and tumor number on prognosis differed between the two groups. These findings emphasize the importance of identifying eMS as a separate subtype in future prognostic studies.

Regarding treatment options, surgery, chemotherapy, radiotherapy (RT), and hematopoietic stem cell transplantation (HSCT) are all available for patients with MS (23). However, there are no agreed guidelines for MS due to its rarity, neither criteria for local therapy and chemotherapy nor indications for HSCT. For either isolated MS or MS that is synchronous with AML, intensive anti-AML chemotherapeutic protocols are currently recommended as systemic therapy, and HSCT is recommended as consolidation therapy (5, 19). In our study, half of the patients underwent chemotherapy, nearly a quarter received RT, more than one in eight patients underwent surgery, and the number of those who received HSCT was unavailable. Although the NCDB-based study included information on individuals who received HSCT, our SEER-based study included more patients who received the other three treatment types (10). Regarding local treatment, several studies have revealed that surgery or RT can shrink tumors, relieve local symptoms, and sometimes aid in diagnosis; however, they do not affect survival (24–26). In addition, as MS molecular mechanism’s understanding improves, targeted therapy is also on the horizon (27, 28).

Although extensive studies have been conducted on MS prognosis, the published findings are controversial, and various prognostic factors have been identified in different studies (17, 29, 30). Patients with MS have a dismal prognosis, with a reported median OS time of <12 months (10, 21). This study found that the median OS times for MS and eMS were 9 and 10 months, respectively. We also revealed that the hMS prognosis was significantly worse, with a median OS time of 5 months. Our study demonstrated that eMS survival outcomes varied significantly with age, primary site, first primary tumor, and chemotherapy. A disparity in OS according to sex and race was observed in another NCDB-based study but not in this study (31). In line with the finding that patients with AML secondary to MS have a better prognosis than those with non-MS AML, patients with eMS as the first primary malignancy have a higher survival rate. This may have resulted from a lead-time advantage in patients with MS, indicating delayed AML development (5, 9). Chemotherapy has long been recognized as an independent prognostic factor, and patients who received chemotherapy had a significantly higher survival rate than those who did not (31). Two NCDB-based studies provided a more thorough analysis of chemotherapy’s effect on the prognosis of patients with MS. Lontos et al. argued that combining chemotherapy with surgery and RT did not improve survival in isolated MS. Goyal et al. found that early chemotherapy was associated with a higher mortality rate among the elderly but had no effect on survival in younger patients (10, 31).

The nomogram can assist clinicians in assessing patient prognosis by converting the miscellaneous COX regression analysis results into a visual predictive model. Although numerous cancer nomograms have been constructed using the SEER database, no nomogram for MS has been reported (32). Our study constructed eMS nomograms for OS and CSS with four independent prognostic factors (age, primary site, first primary tumor, and chemotherapy). Our models had excellent discriminating and calibration abilities and potential clinical utility in the training and validation cohorts. To our knowledge, there is no risk stratification model for patients with MS. Clinicians can predict the 1-, 3-, and 5-year survival rates of patients with eMS and categorize them into low- or high-risk groups using the total points of the nomograms in this study.

### Limitations

This study has numerous limitations, similar to those found in other studies based on retrospective datasets, including NCDB and SEER (9, 10). First, the SEER database was based on the US population, which may have limited our findings’ generalizability. Second, although HSCT has been shown to affect MS prognosis, it was not incorporated into our clinical characteristics-based model because the information was unavailable in the SEER database (33). A further limitation on the predictive significance of chemotherapy is that the SEER database only provides information on whether patients underwent chemotherapy, not detailed individual chemotherapy regimens. As MS receives more attention, we will have additional information from single-center institutions or multi-center collaborative groups to help address this issue. Recently, oncology researchers have focused on imaging and genetic characteristics’ impact on prognosis (34). The PET/CT potential utility in monitoring and assessing therapeutic response in MS has been emphasized by Lee et al. (35). Although we have gained further insight into the cytogenetic and molecular abnormalities of MS, such as chromosomal abnormalities like inv (16) and t (8, 21), and mutations in NPM1 and FLT3, the prognostic impact of genetic features remains poorly understood due to the small sample size (16, 36, 37). Therefore, another limitation is that it lacks information on the patients’ imaging, cytogenetic, and molecular features. As a result, we could not investigate these features’ predictive significance even with the sufficiently large sample size.

### Conclusion

Using the SEER database, we updated the information on patients with MS and compared the clinical features and prognostic markers between the eMS and hMS groups, supporting the recommendation of distinguishing patients with eMS from those with hMS in future studies. Furthermore, our research developed and validated novel nomograms exclusively for patients with eMS. These models may assist clinicians in predicting overall and cancer-specific survival rates. In future studies, patients will benefit from new prognostic models that combine the clinical features with MS genomic, transcriptomic, and metabolomic features.

## Data availability statement

Publicly available datasets were analyzed in this study. This data can be found here: https://seer.cancer.gov/data-software/.

## Author contributions

XWZ, ZX, and XHZ made major contributions to the design and data collection of this study. ZX, XHZ, ZL, HW, and MQ participated in the data analysis, graphing, and compilation of the results. XWZ, ZX, and XHZ wrote the manuscript. ZL, HW, and MQ revised the manuscript. All authors contributed to the article and approved the submitted version.

## Funding

This work was supported by the National Natural Science Foundation of China (82141125), the Cyrus Tang Foundation (ZSBK0070), Shanghai Fudan University Education Development Foundation (ZSBK0046), Shanghai Municipal Committee of Science and Technology (21Y31900302), and Shanghai Hospital Development Center (SHDC12019121).

## Acknowledgments

The authors thank the SEER database for providing the platform, the hospital institutions for uploading clinical information, and the staff responsible for maintaining and updating the SEER database. We also thank the developers of the R packages used in our research for making all this work possible. Finally, we would like to thank Editage (www.editage.cn) for English language editing.

## Conflict of interest

The authors declare that the research was conducted in the absence of any commercial or financial relationships that could be construed as a potential conflict of interest.

## Publisher’s note

All claims expressed in this article are solely those of the authors and do not necessarily represent those of their affiliated organizations, or those of the publisher, the editors and the reviewers. Any product that may be evaluated in this article, or claim that may be made by its manufacturer, is not guaranteed or endorsed by the publisher.

## Abbreviations

MS, Myeloid sarcoma; DCA, decision curve analysis; OS, overall survival; CSS, cancer-specific survival; hMS, myeloid sarcoma involving hematopoietic site; eMS, myeloid sarcoma excluding those involving hematopoietic site; AML, acute myeloid leukemia; MDS, myelodysplastic syndrome; MPN, myeloproliferative neoplasm; ML, chronic myelogenous leukemia; eAML, extramedullary acute myeloid leukemia; AIC, Akaike information criterion; AUC, area under the receiver operating curve; APC, annual percentage change; DCA, Decision curve analysis; RT, radiotherapy; HSCT, hematopoietic stem cell transplantation.
