# Supplementary material for: Incidence, clinical characteristics, and prognostic nomograms for patients with myeloid sarcoma: A SEER-based study
Source: Front Oncol. 2022 Aug 18;12:989366. doi: 10.3389/fonc.2022.989366 (PMC9433649; doi:10.3389/fonc.2022.989366)
Supplement: Supplementary file 1 [file Image_1.pdf]

1.1 Supplementary Tables

Supplementary Table S1. Patient prognosis according to primary sites.

				OS			CSS		
Site	Number (%)	Age, y mean(SD)	Dead (%)	Median m	1-year (%)	3-year (%)	Median m	1-year (%)	3-year (%)
All	694(100)	56.2(22.3)	498 (71.8)	9	43.5	31.3	11	48.0	37.7
hMS	86(12.4)	61.7(19.7)	71 (82.6)	5	32.0	20.1	5	34.0	26.0
eMS	608(87.6)	55.5(22.5)	427(70.2)	10	45.1	32.9	13	50.0	39.4
st	247(35.6)	55.0(17.0)	185 (74.9)	7	37.1	27.6	9	41.8	34.4
s/b	90(13.0)	60.7(25.0)	57 (63.3)	18	56.5	36.0	31	60.8	44.4
bo	43(6.2)	51.1(22.5)	33 (76.7)	8	43.6	21.4	9	49.7	26.8
ns	21(3.0)	55.0(17.0)	17 (81.0)	2	20.8	15.6	3	28.9	21.6
h/n	51(7.3)	49.6(26.7)	33 (64.7)	12	47.8	39.9	34	54.1	48.2
ds	65(9.4)	49.3(18.9)	39 (60.0)	21	55.6	46.7	29	58.7	49.3
c/m	24(3.5)	52.8(23.3)	17 (70.8)	10	38.8	28.3	12	45.6	33.2
rs	41(5.9)	49.3(18.9)	25 (61.0)	37	64.6	50.7	38	66.6	55.3
k/b/r	26(3.7)	57.5(13.6)	21 (80.8)	8	46.2	30.8	18	51.3	38.5

OS, overall survival; CSS, cancer-specific survival; m, month; st, soft tissue; s/b, skin/breast; b, bone; ns, nervous system; h/n, head/neck; ds, digestive system; c/m, cardiopulmonary/mediastinum; rs, reproductive System; k/b/r, kidney/bladder/retroperitoneum

Supplementary Table S2. Clinical characteristics and survival outcomes of MS patients under 15 years old.

Characteristic	<15y N(%)	⩾15y N(%)
Sum	47(6.8)	661(93.2)
Age at diagnosis (years)		
Mean (SD)	5.00 (4.81)	60.0 (18.0)
Median [Min, Max]	4.0 [0, 14.0]	64 [15.0,96.0]
Number		

≥3	0 (0)	113 (17.5)
1	34 (72.3)	273 (42.2)
2	13 (27.7)	261 (40.3)
1st primary tumor		
No	6 (12.8)	294 (45.4)
Yes	41 (87.2)	353 (54.6)
Site		
hMS	3 (6.4)	83 (12.8)
eMS	44 (93.6)	564 (87.2)
st	15 (31.9)	232 (35.9)
s/b	7 (14.9)	83 (12.8)
bo	5 (10.6)	38 (5.9)
ns	1 (2.1)	20 (3.1)
h/n	9 (19.1)	42 (6.5)
ds	3 (6.4)	62 (9.6)
c/m	2 (4.3)	22 (3.4)
rs	2 (4.3)	39 (6.0)
k/b/r	0 (0)	26 (4.0)
Chemotherapy		
No/Unknown	12 (25.5)	335 (51.8)
Yes	35 (74.5)	312 (48.2)
Status		
Alive	33 (70.2)	163 (25.2)
Dead	14 (29.8)	484 (74.8)
Median(m)	NA	8
1-year OS(%)	81.0	40.8
3-year OS(%)	67.4	28.8

y, years; N, number of patients; st, soft tissue; s/b, skin/breast; b, bone; ns, nervous system; h/n, head/neck; ds, digestive system; c/m, cardiopulmonary/mediastinum; rs, reproductive System; k/b/r, kidney/bladder/retroperitoneum; m, month; OS, overall survival.

Supplementary Table S3. Baseline characteristics of patients with eMS in the training and validation groups.

Characteristic	Validation Cohort N(%)	Train Cohort N(%)	Total N(%)	P.Value
Sum	183(30)	425(70)	608(100)	
Age at diagnosis (years)				0.999
Mean (SD)	55.3 (23.3)	55.6 (22.2)	55.5 (22.5)	
Median [Min, Max]	62.0 [0, 93.0]	60.0 [0, 96.0]	61.0 [0, 96.0]	
Age group (years)				0.587
<40	44 (24.0)	96 (22.6)	140 (23.0)	
40-59	41 (22.4)	112 (26.4)	153 (25.2)	
≥60	98 (53.6)	217 (51.1)	315 (51.8)	
Year of diagnosis				0.592
2000-2009	64 (35.0)	160 (37.6)	224 (36.8)	
2010-2018	119 (65.0)	265 (62.4)	384 (63.2)	
Sex				0.740
Female	75 (41.0)	182 (42.8)	257 (42.3)	
Male	108 (59.0)	243 (57.2)	351 (57.7)	
Race				0.387
Asian	18 (9.8)	29 (6.8)	47 (7.7)	
White	146 (79.8)	344 (80.9)	490 (80.6)	
Others	19 (10.4)	52 (12.2)	71 (11.7)	
Marital.status				0.382
Single	44 (24.0)	107 (25.2)	151 (24.8)	
Married	100 (54.6)	204 (48.0)	304 (50.0)	
Widowed	14 (7.7)	35 (8.2)	49 (8.1)	
Others	25 (13.7)	79 (18.6)	104 (17.1)	
Site				0.692
setA	23 (12.6)	41 (9.6)	64 (10.5)	
setB	89 (48.6)	208 (48.9)	297 (48.8)	
setC	58 (31.7)	148 (34.8)	206 (33.9)	
setD	13 (7.1)	28 (6.6)	41 (6.7)	
Number				0.273

≥3	31 (16.9)	63 (14.8)	94 (15.5)	
1	86 (47.0)	179 (42.1)	265 (43.6)	
2	66 (36.1)	183 (43.1)	249 (41.0)	
1st primary tumor				0.376
No	74 (40.4)	190 (44.7)	264 (43.4)	
Yes	109 (59.6)	235 (55.3)	344 (56.6)	
Surgery				0.999
No/Unknown	157 (85.8)	366 (86.1)	523 (86.0)	
Yes	26 (14.2)	59 (13.9)	85 (14.0)	
Radiation				0.514
No/Unknown	138 (75.4)	308 (72.5)	446 (73.4)	
Yes	45 (24.6)	117 (27.5)	162 (26.6)	
Chemotherapy				0.620
No/Unknown	90 (49.2)	220 (51.8)	310 (51.0)	
Yes	93 (50.8)	205 (48.2)	298 (49.0)	

N, number of patients; setA, nervous system and bone; setB, cardiopulmonary/mediastinum , kidney/bladder/retroperitoneum and soft tissue; setC, skin/breast, head/neck, and digestive system; setD, reproductive system.

Supplementary Table S4. Univariate COX regression analyses of OS and CSS in the training cohort

Characteristics	OS			CSS		
	HR	95%CI	P.Value	HR	95%CI	P.Value
Age group (years)						
<40	Ref			Ref		
40-59	2.02	1.37-2.98	<0.001	1.66	1.09-2.52	0.019
≥60	4.70	3.31-6.67	<0.001	3.82	2.64-5.52	<0.001
Year of diagnosis						
2000-2009	Ref			Ref		
2009-2018	1.09	0.86-1.37	0.482	1.08	0.83-1.40	0.562
Sex						
Female	Ref			Ref		
Male	1.19	0.94-1.49	0.144	1.11	0.86-1.43	0.436

Race						
Asian	Ref			Ref		
White	1.60	0.97-2.66	0.068	1.79	1.00-3.20	0.051
Others	1.57	0.87-2.83	0.134	1.53	0.77-3.04	0.221
Marital.status						
Single	Ref			Ref		
Married	1.78	1.31-2.42	<0.001	1.58	1.13-2.2	0.007
Widowed	4.57	2.96-7.05	<0.001	3.70	2.29-5.99	<0.001
Others	2.03	1.41-2.93	<0.001	1.70	1.14-2.55	0.010
Number						
≥3	Ref			Ref		
1	0.46	0.33-0.64	<0.001	0.43	0.30-0.61	<0.001
2	0.66	0.48-0.91	0.010	0.59	0.42-0.83	0.002
1st Primary Tumor						
No	Ref			Ref		
Yes	0.52	0.42-0.66	<0.001	0.53	0.41-0.68	<0.001
Site						
SetA	Ref			Ref		
SetB	0.69	0.47-1.00	0.050	0.65	0.43-0.97	0.033
SetC	0.46	0.31-0.69	<0.001	0.44	0.29-0.68	<0.001
SetD	0.33	0.18-0.60	<0.001	0.29	0.15-0.57	<0.001
Surgery						
No/Unknown	Ref			Ref		
Yes	0.65	0.46-0.91	0.011	0.62	0.42-0.92	0.016
Radiation						
No/Unknown	Ref			Ref		
Yes	0.95	0.74-1.23	0.715	0.93	0.70-1.23	0.588
Chemotherapy						
No/Unknown	Ref			Ref		
Yes	0.46	0.36-0.58	<0.001	0.49	0.38-0.64	<0.001

setA, nervous system and bone; setB, soft tissue, cardiopulmonary/mediastinum and kidney/bladder/retroperitoneum; setC, skin/breast, head/neck, and digestive system; setD, reproductive system.

1.2 Supplementary Figures

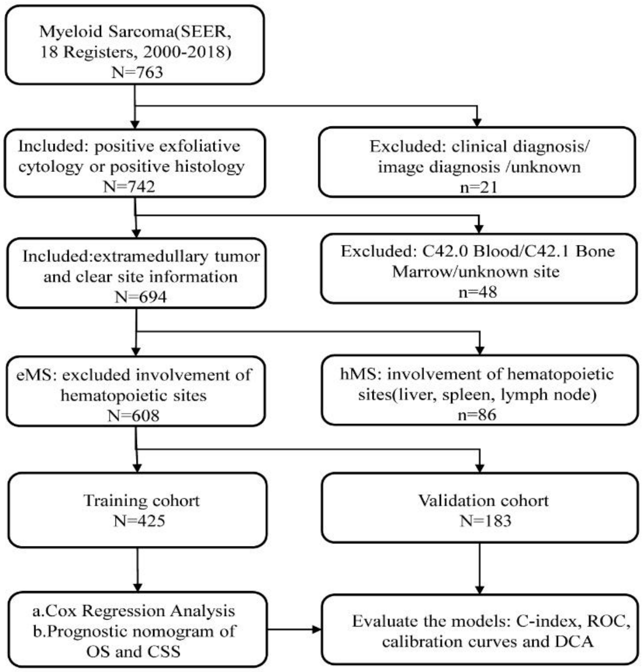

Supplementary Figure S2. Flowchart for inclusion and exclusion of patients and subsequent analysis process.

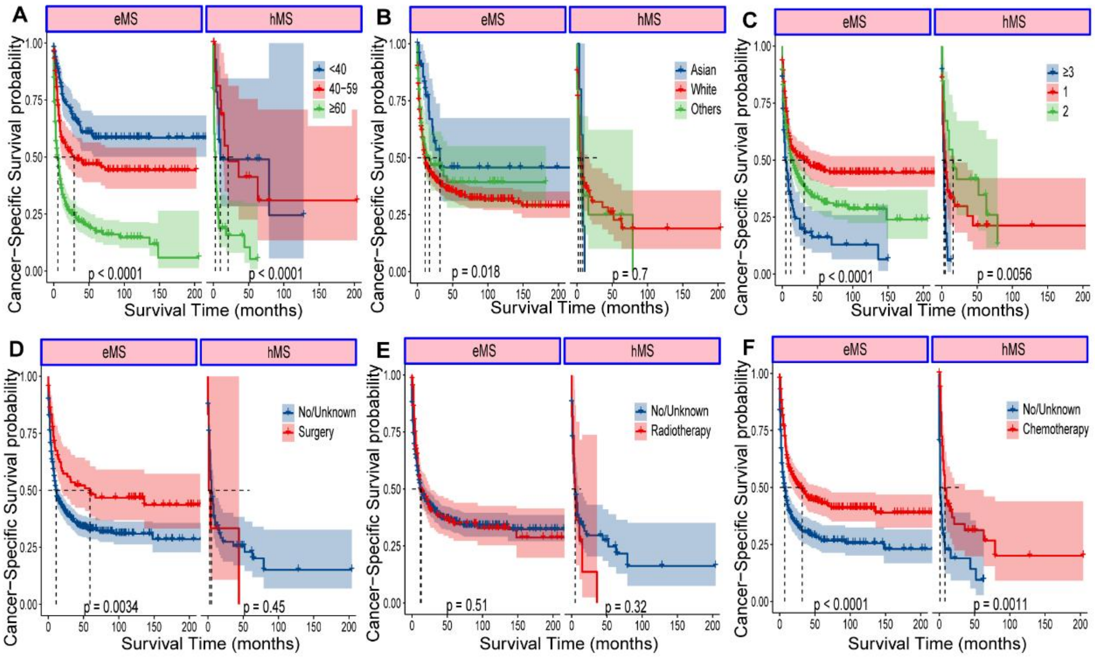

Supplementary Figure S2. Kaplan–Meier analysis of cancer-specific survival (CSS) in eMS and hMS, respectively. Kaplan–Meier survival curves of CSS for patients with eMS or hMS stratified by age **(A)**, race **(B)**, number of tumors **(C)**, surgery **(D)**, radiotherapy **(E)** and chemotherapy **(F)**.

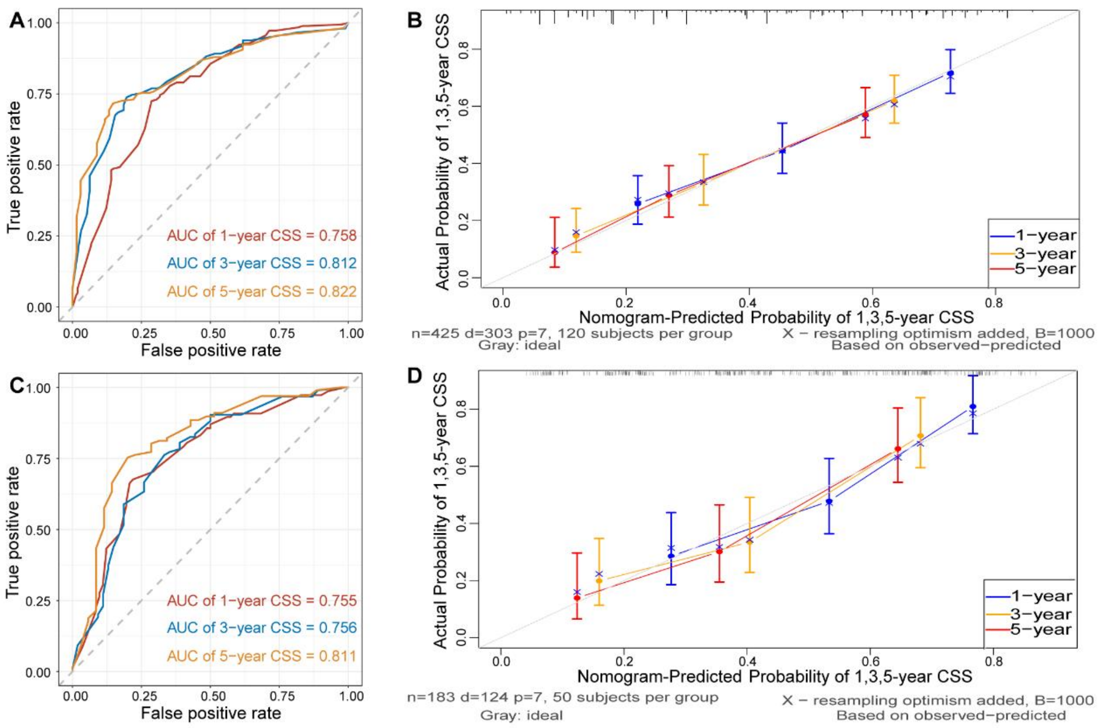

Supplementary Figure S3. Receiver operating characteristic (ROC) and calibration curves of the nomogram for CSS. ROC curves were plotted to evaluate the performance of the model to discriminate between patients with different CSS outcomes (alive or dead), quantified by calculating the area under the ROC curve (AUC). The AUC of nomogram for predicting 1-, 3-, and 5-year CSS were 0.758, 0,812 and 0.822 in the training cohort (A) and 0.755, 0.756, and 0.811 in the validation cohort (C). Calibration curves were plotted to evaluate the accuracy of the nomogram model. The horizontal axis represents the survival rate predicted by the model, and the vertical axis represents the actual survival rate. The diagonal line represents the ideal situation where the predicted and actual survival rates consist, and the blue, orange, and red lines represent the model's predicted and actual survival rates for 1-year, 3-year, and 5-year CSS, respectively. Calibration curves of the nomogram for predicting 1-, 3-, and 5-year CSS in the training cohort (B) and in the validation cohort (D).

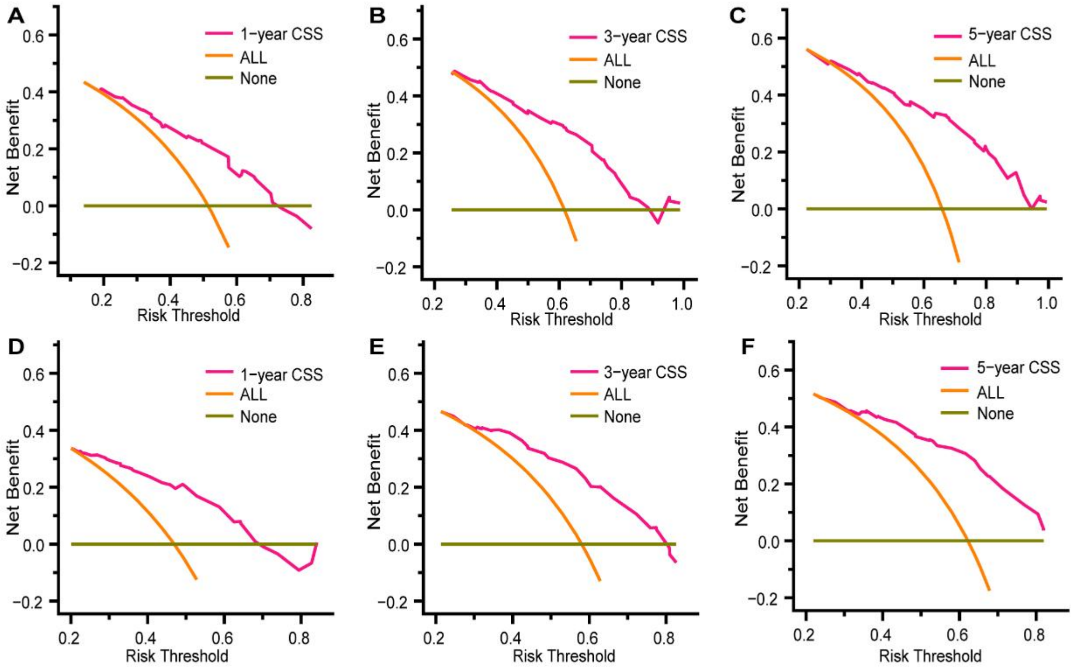

Supplementary Figure S4. Decision curve analysis (DCA) of the nomogram for predicting 1-, 3- and 5- year CSS rates in patients with eMS in the training cohort **(A-C)** and the validation cohort **(D-F)**. DCA was used to evaluate the clinical utility of the nomogram by calculating the net benefits of the model under different thresholds. The x-axis represents threshold probability, and the y-axis represents net benefit. The horizontal dark green line represents no cancer-specific deaths occurring, and the orange line represents all patients died attributing to this cancer. The pink line represents our nomogram model and when it is maintained above the dark green and orange line mentioned above, the net benefit value of the model is positive, which implies that our model has good clinical utility.

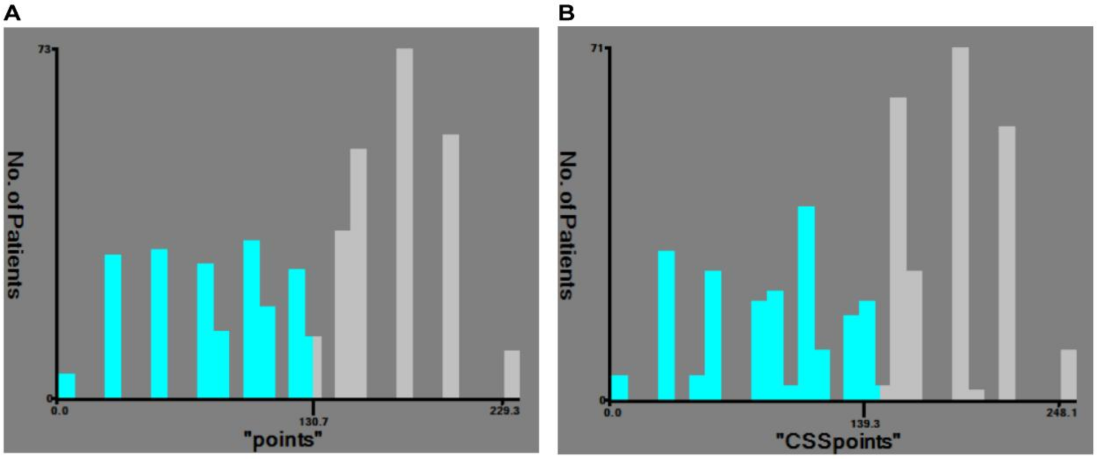

Supplementary Figure S5. The best cutoff points for risk stratification generated by X-tile software. The best cutoff points were 130.7 in the nomogram of OS **(A)** and 139.3 in the nomogram of CSS **(B)**. The x-axis represents the total points of patients according to the nomogram, and the y-axis represents the number of patients who have the same total points. The best cut-off values of total

points generated by the X-tile can divide patients into two groups with the most significant different survival outcomes.
